# Variation in resistance to multiple pathogen species: anther smuts of *Silene uniflora*

**DOI:** 10.1002/ece3.346

**Published:** 2012-08-13

**Authors:** Erin Chung, Elsa Petit, Janis Antonovics, Amy B Pedersen, Michael E Hood

**Affiliations:** 1 Department of Biology, Amherst CollegeAmherst, Massachusetts; 2 Department of Biology, University of VirginiaCharlottesville, Virginia; 3 Centre of Immunity, Infection and Evolution, Institute of Evolutionary Biology, School of Biological Sciences, University of EdinburghEdinburgh, EH9 3JT, UK

**Keywords:** Co-infection, general resistance, host specificity, *Microbotryum*, multi-parasitized hosts

## Abstract

The occurrence of multiple pathogen species on a shared host species is unexpected when they exploit the same micro-niche within the host individual. One explanation for such observations is the presence of pathogen-specific resistances segregating within the host population into sites that are differentially occupied by the competing pathogens. This study used experimental inoculations to test whether specific resistances may contribute to the maintenance of two species of anther-smut fungi, *Microbotryum silenes-inflatae* and *Microbotryum lagerheimii*, in natural populations of *Silene uniflora* in England and Wales. Overall, resistance to the two pathogens was strongly positively correlated among host populations and to a lesser degree among host families within populations. A few instances of specific resistance were also observed and confirmed by replicated inoculations. The results suggest that selection for resistance to one pathogen may protect the host from the emergence via host shifts of related pathogen species, and conversely that co-occurrence of two species of pathogens may be dependent on the presence of host genotypes susceptible to both.

## Introduction

Disease ecology has progressed tremendously in the last 30 years through a focus on simplified one-host one-pathogen models. However, recent studies have begun to emphasize the importance of more complex pathogen communities that normally infect single individuals or single host species in nature (Petney and Andrews 1998; Read and Taylor 2001; Lello et al. 2004; Pedersen and Fenton 2007; Fenton 2008; Rigaud et al. 2010). For example, multiple pathogen species have been shown to interact within individual hosts, resulting in compounded negative effects on individual host fitness (Lello et al. 2004) and such multiple infection may be a primary driver of virulence evolution (van Baalen and Sabelis 1995; Frank 1996). These pathogen interactions within an individual host are thought to occur either directly or indirectly, as a result of shared host resource or host immune-mediated interactions. Recent research from human co-infection studies suggest that most interactions between pathogens are synergistic, that is, the presence of one pathogen species increases the presence of the other, for example, resulting in higher parasite abundances in co-infected individuals, and consequently greater negative effects for health (Griffiths et al. 2011). However, factors allowing the long-term co-occurrence of multiple pathogen species on a single host species are poorly understood from both the theoretical and empirical perspective, limiting our ability to predict the overall threat of co-infection for host health and to anticipate the impact of controlling one pathogen on other members of the pathogen community.

Based on analogies from community ecology, a susceptible host individual can be viewed as a set of heterogeneous resource patches, where different parts of the host (e.g., gastrointestinal tract vs. blood in animals, leaves vs. roots in plants, etc.) might provide niches that are sufficiently discrete to reduce competition between spatially separated pathogens (Holt and Dobson 2006; Pedersen and Fenton 2007). Recent research has suggested that many parasites are likely to interact indirectly with other species at the level of the individual host, through segregated host resources (Griffiths et al. 2011). However, closely related pathogens, such as members of the same genus, are more likely to occupy identical niches, and their occurrence on the same host species is less easily explained by resource partitioning. For example, combinations of several malaria-causing *Plasmodium* species have been observed in sympatry among human and avian populations, even co-infecting individual hosts and these different *Plasmodium* species have been shown to target different red blood cell classes (McQueen and McKenzie 2004).

Another explanation of the persistence of closely related pathogen species on a single host species may be genetic polymorphisms for resistance to a specific pathogen (Dwyer et al. 1990; Aparicio et al. 2004) that allows co-occurrence of the pathogen at the population level, but not at the level of host individuals. It is well known that gene-for-gene systems, as well as specific induced immunity, can lead to the maintenance of multiple genotypes of a pathogen species on a single population or species of host (Cox 2001; Gruner et al. 2009). However, it is largely unknown to what extent such specificities in resistance occur at the within pathogen species level or how these will translate to among pathogen species (Heath 1981).

The interactions between species-specific resistance and the maintenance of multiple-related pathogen species on a single host species are not well understood because there are few theoretical models or empirical studies (Roode et al. 2003; Aparicio et al. 2004). Some basic assumptions of trait evolution would apply to resistance to multiple pathogens species, including whether multiple species-specific resistances are genetically correlated. In addition, their long-term co-evolutionary dynamics is likely to be limited by the strength of any associated resistance costs to the individual host and the complexity of the pathogen community (Parker 1992; Antonovics 1994). More generally, studies on resistance to multiple pathogen species have been very limited (Leimu and Fischer 2008; Wisser et al. 2011), and results have shown either positive, negative, or the lack of a correlation across host genotypes. In other non-pathogenic systems, the evolution of structural defenses against herbivores in plants and parasitoids in insects function most often as general protection against groups of similar enemies (Boulétreau and Wajnberg 1986; Parker 1992).

Anther-smut disease, caused by fungal pathogens in the genus *Microbotryum*, occurs frequently on plants in the Caryophyllaceae (Hood et al. 2010) and provides a suitable model for studying host–pathogen interactions in natural systems (Bernasconi et al. 2009). Although there is usually a high degree of host-species specificity in the pathogens (Le Gac et al. 2007a), multiple *Microbotryum* species sometimes occur on a single host species. For example, *Silene vulgaris* in alpine regions of Europe harbors three *Microbotryum* species, *M. silenes-inflatae*, *M. lagerheimii*, and *M. violaceo-irregulare* (Kemler et al. 2006; Denchev 2007), and there are sites with complete sympatry of pathogen species within a population of one host species (Bucheli et al. 2000). It is unlikely that these *Microbotryum* species coexist because they occupy different morphological niches within the host individuals because anther-smut pathogens reside within a very limited region of the plant apical meristems and only produce spores in anthers of the flowers (Day 1980; Audran and Batcho 1982; Schäfer et al. 2010). Thus, pathogen-specific resistance is a possible alternative explanation of the occurrence of multiple *Microbotryum* species on a single host species. However, little is known about the specificity of resistance in any *Silene*-*Microbotryum* system. Studies have shown that there is substantial genetic variation for resistance in host populations, and it can sometimes have a simple genetic basis with a bimodal segregation, as in *S. vulgaris* (Cafuir et al. 2007), or be more continuously variable, as in *S. latifolia* (Alexander and Antonovics 1995; Biere and Antonovics 1996). Where variation in pathogen infectivity and host-genotype by pathogen-genotype interaction effects have been measured, sufficient variation in the pathogen infectivity, so that it could reflect local adaptation, was not observed (Carlsson-Graner 1997; Kaltz and Shykoff 2002).

Here, we present a study of anther-smut disease on *Silene uniflora* (= *S. maritima*), a close relative of *S. vulgaris*, which also harbors populations of *M. silenes-inflatae* and *M. lagerheimii*. *Silene uniflora* is endemic to coastal regions of central and northern Europe, and its populations are disjunct from diseased populations of *S. vulgaris* in the Alps. We describe the occurrence of *M. silenes-inflatae* and *M. lagerheimii* in populations of *S. uniflora* in England and Wales. We then use experimental inoculations to determine whether pathogen-specific resistances may contribute to the maintenance of multiple *Microbotryum* species *S. uniflora* by quantifying family-level and population-level variation and covariation for resistance to these pathogens.

## Material and Methods

### Study system

**Figure 1:**
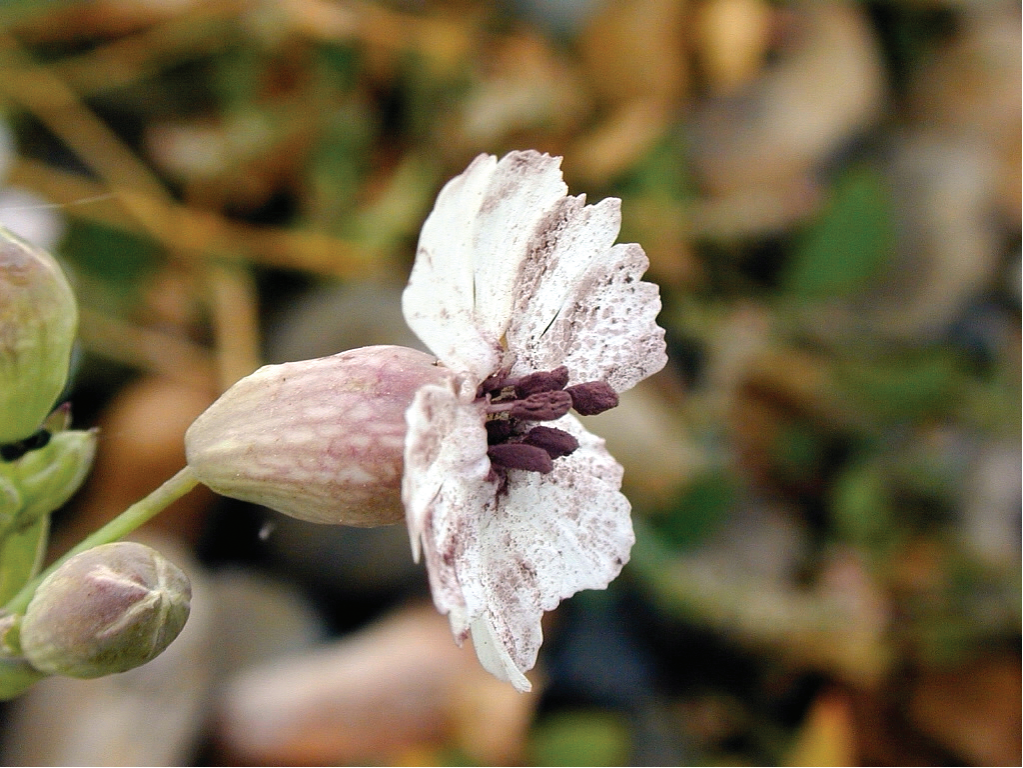
Anther-smut disease of *Silene uniflora*, caused by the fungi in the genus *Microbotryum*. The teliospores of the fungal pathogen replace the pollen of the plant and are carried to other plants by insect pollinators.

Fungi in the genus *Microbotryum* (Basidiomycota) cause anther-smut disease on many perennial species in the Caryophyllaceae (Hood et al. 2010). Anther-smut disease results in the pathogen replacing pollen in the host's anthers with fungal spores (i.e., teliospores) (Fig. 1), which are then transmitted to other plants by insect pollinators. The pathogen is generally found in all flowers of an infected plant and the disease results in the abortion of female structures, such that infected plants are sterilized. The disease cycle and life history, which are general across *Microbotryum* anther smuts, are summarized in Giraud et al. (2008). There is little effect of infection upon individual host mortality, and the pathogen can persist for many years as it overwinters inside the host. The existence of host-species specificity among *Microbotryum* isolates from different host species has been known since the early 1900s (Goldschmidt 1928), but only recently have detailed morphological studies and molecular phylogenies helped to confirm the existence of such multiple species-specific *Microbotryum* lineages (Vánky 2004; Le Gac et al. 2007a; Denchev et al. 2009), and some, including those studied here, have been given species names (Denchev 2007; Denchev et al. 2009).

The host *Silene uniflora* (= *Silene maritima*) harbors populations of *Microbotryum lagerheimii* and *Microbotryum silenes-inflatae*. Within *Microbotryum* on European *Silene*, based on DNA sequences of three single copy genes, these two species are highly divergent, and in separate clades containing species that are specific to other hosts (Le Gac et al. 2007a; note that the species in this article are referred to as “MvSv1” and “MvSv2,” respectively). The two species are cross-compatible, but their hybrids showed a reduced ability to infect relative to hybrids between more closely related species (cf. Gac et al. 2007b). High elevation populations of the closely related plant *Silene vulgaris* (Runyeon and Prentice 1997), also harbor *M. silenes-inflatae* and *M. lagerheimii*, as well as other *Microbotryum* species (Bucheli et al. 2000; Kemler et al. 2006; Denchev 2007; Le Gac et al. 2007a; Lutz et al. 2008). Inoculation studies have also demonstrated the potential for co-infections by *M. silenes-inflatae* and *M. lagerheimii* of individual plants of *S. vulgaris* (Gold et al. 2009).

*Silene uniflora* is a gynodioecious long-lived perennial herb found mostly in coastal habitats in central and northern Europe. A small number of inland populations exist in the United Kingdom, primarily associated with high elevation sites or in heavy metal contaminated soils (Marsden-Jones and Turril 1957). Prior studies of anther-smut disease on this host are very limited (Evans and Wilson 1971).

### Field surveys and collections

**Figure 2:**
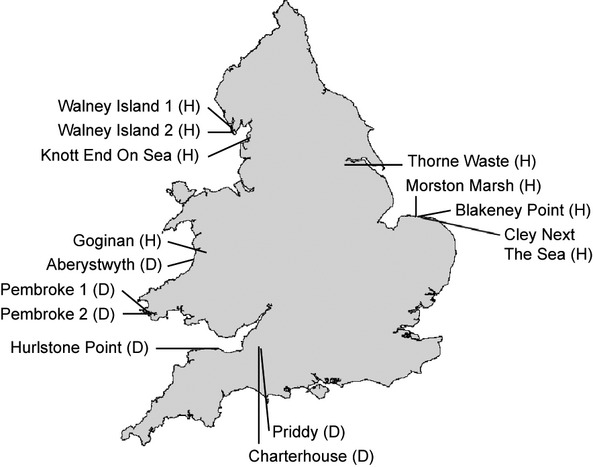
Sampling localities and the occurrence of anther-smut disease on *Silene uniflora* in England and Wales. Details of localities are given in Table 1. Populations free of disease (Healthy) and populations where disease was found (Diseased) are indicated by “H” and “D,” respectively.

*Silene uniflora* populations in England and Wales were identified using locations noted in the literature, herbarium records, and online resources such as Wild About Britain (http://www.wildaboutbritain.co.uk). Fourteen populations were surveyed in 2008, at which time seeds were collected by common female parent (Table 1; Fig. 2). Selecting individual plants was conducted haphazardly, but at well-spaced intervals throughout each population to provide representative collections. Seeds of *S. uniflora* were collected as maternal half-sib families by taking seeds from one plant where the source of pollen was likely to include multiple plants. As individuals of *S. uniflora* typically grow as large mats that can become intertwined in high-density populations, care was taken to trace flowering stems back to a common tap root to ensure that seeds from separate maternal sources were not mixed. For most sites, more than 10 families were sampled for seeds and multiple capsules were collected for each family to maximize the seed availability (Table 1).

**Table 1:** Population data for collections of *Silene uniflora* seeds and *Microbotryum* samples

Location name	Description of location and year visited	Latitude, longitude	Number of healthy individuals (families sampled for seeds)	Number of diseased individuals (individuals sampled for disease)	*Microbotryum* species
Thorne Waste* (Yorkshire, England)	Inland bog	+53.40825, −0.20176	250 (12)	0 (0)	
Cley Next The Sea* (Norfolk, England)	Shingle beach	+52.965866, +1.042957	100–200 (12)	0 (0)	
Blakeney Point* (Norfolk, England)	Shingle beach	+52.963131, +0.989013	1000 (12)	0 (0)	
Morston Marsh (Norfolk, England)	Coastal marsh	+52.96317, +0.97074	80 (10)	0 (0)	
Knott End On Sea (Lancashire, England)	Coastal meadow	+53.92850, −2.99431	30 (0)	0 (0)	
Walney Island 1* (Cumbria, England)	Shingle beach	+54.10965, −3.26817	150 (12)	0 (0)	
Walney Island 2* (Cumbria, England)	Shingle beach	+54.06206, −3.22281	250 (12)	0 (0)	
Goginan (Ceredigion, Wales)	Inland near mine	+52.42836, −3.91877	120 (7)	0 (0)	
Aberystwyth* (Ceredigion, Wales)	Coastal cliff	+52.42336, −4.08432	400 (14)	30–60 (25)	*M. s.-i*.
Pembrokeshire Coast 1* (Wales)	Coastal cliff	+51.66542, −5.07025	150 (7)	3 (3)	*M. s.-i*.
Pembrokeshire Coast 2* (Wales)	Coastal cliff	+51.66612, −5.08022	50–100 (7)	5 (5)	*M. s.-i*.
Hurlstone Point* (Somerset, England)	Coastal cliff	+51.23113, −3.57748	80 (14)	3 (3)	*M. s.-i*.
Charterhouse* (Somerset, England)	Inland mine	+51.30109, −2.70838	1000 (17)	500 (25)	*M. s.-i./M. l*.
Priddy* (Somerset, England)	Inland mine	+51.25927, −2.64991	80 (8)	4 (3)	*M. l*.

The site names, descriptions, year of sampling, and GPS coordinates are given. Approximate numbers of healthy and diseased individuals are listed with the number of plants sampled families for seeds and fungal spores, respectively, given in parentheses. Asterisks indicate source populations for seeds used in the inoculation experiment. Identified *Microbotryum* species are abbreviated as *M. s.-i*. for *M. silenes-inflatae* and *M. l*. for *M. lagerheimii*.

The latitude and longitude of each locality were recorded, as well as estimates of the number of healthy and diseased host plants (Table 1). To minimize cross-contamination in the field, *Microbotryum* samples were collected as mature but unopened flower buds from diseased plants rather than as open flowers.

PCR-RFLP was used to assign field-collected specimens to either *M. silenes-inflatae or M. lagerheimii*. Following DNA extraction from spore-filled anthers using the Chelex method of Bucheli et al. (2001), the internal transcribed spacer (ITS) region of the nuclear ribosomal RNA genes was amplified by PCR. The PCR product was digested by the *Hha*I restriction enzyme, where the restriction digest banding pattern differentiates *M. lagerheimii* from *M. silenes-inflatae*. ITS primers were designed to be specific to *Microbotryum* to avoid amplification of plant DNA: forward primer (5′ to 3′) CTGTTTAACCAGGGCGTGAC and reverse primer (5′ to 3′) TGATCTCGAAGGTTAGGATGC.

### Variation in physiological resistances

To assess variation in physiological resistance, which is the prevention of disease following exposure to the pathogen, in *S. uniflora*, families were chosen with sufficient numbers of seeds to inoculate 30–50 individual plants per family with four pathogen genotypes, two from each of the two *Microbotryum* species applied to each host singly (see below). Families with too few seedlings to receive all four treatments were instead inoculated with two pathogen genotypes, one randomly chosen from each of the two *Microbotryum* species. Families from five populations free of disease and six populations where disease was present were used in the inoculation study (Table 1). Seeds were surface-sterilized in a dilute solution of sodium hypochlorite and alcohol and germinated in 150 × 15 mm Petri dishes containing 1/10th strength Murashige and Skoog Basal Salt Mixture (Sigma-Aldrich, St. Louis, MO) and 1% agar. After 10 days of incubation at 15°C, when most seedlings had fully expanded their cotyledons, 4 μL of inoculum was applied to the apical meristem as a suspension of 1400 fungal spores in water plus a surfactant (as in Hood 2003). In the anther-smut disease system, resistance variation assessed by experimental inoculation has been shown to strongly predict rates of disease transmission in the field (Alexander et al. 1993).

Seedlings from each family were randomly assigned to receive an inoculation with a pathogen isolate from each of the following source populations: *M. lagerheimii* from Charterhouse, *M. lagerheimii* from Priddy, *M. silenes-inflatae* from Pembroke Coast 1 (Table 1), and *M. silenes-inflatae* from Dunwich. The Dunwich population from Suffolk, England (latitude: +52.280293, longitude +1.634146) was obtained in 2007 as part of a separate study. Pathogen isolates were each obtained from a single diseased flower bud, which almost invariably represents a single genotype (Baird and Garber 1979; López-Villavicencio et al. 2007), sampled at random from the respective populations. For each of the pathogen genotypes, spore germination rates were confirmed to be greater than 90% by examining growth on potato dextrose agar after 24 h at 22°C.

After further incubating the inoculated seedlings at 15°C for 3 days, they were transplanted into 3.8-cm diameter Cone-tainers (Stueweand Sons Inc., Tangent, OR) filled with a soil mixture as described in Cafuir et al. (2007) and grown in the greenhouse under long-day lights (16 h). The experiment was established in winter months, and greenhouse environmental controls were set to a daytime maximum temperature of ca. 20°C and a nighttime minimum temperature between 10 and 15°C. These conditions were determined to be favorable for disease development on *S. uniflora* based on preliminary studies (data not shown). The positions of plants in the greenhouse were completely randomized across treatments and populations.

Upon flowering, each plant was scored as diseased or healthy, based on the presence or absence of fungal spores in the anthers. Plants with diseased flowers were removed after being scored to minimize cross-contamination. Plants that appeared healthy in the first open flower were maintained for two additional weeks separately from the rest of the experimental plants to determine if subsequent flowers were infected, which happened very rarely.

Only flowering plants were assessed for disease and included in the statistical tests. Approximately 7% of the plants failed to flower (see below), and these were equally distributed across the treatments. To minimize the effects of small sample sizes on variance, families with fewer than ten flowering plants were excluded from subsequent analyses. All data on the proportion of plants diseased were arcsine transformed prior to analysis, and numbers of plants were used in weighted analyses. Correlation analyses between infection rates for *M. silenes-inflatae* versus *M. lagerheimii* among *S. uniflora* populations were conducted in SPSS v15 (SPSS, Chicago, IL). Among-family variance component estimates for the correlations and their standard errors were calculated using ASReml v.3 (http://www.vsni.co.uk/software/asreml). Families were identified as outliers in the analysis of correlation between resistance to *M. silenes-inflatae* versus *M. lagerheimii* by calculating *P*-values for Mahalanobis distances (*D*^2^) (Mahalanobis 1936; McLachlan 1999) for each family in SPSS v15. Mahalanobis *D*^2^ measures the distance of a particular case to the multidimensional mean of the remaining distribution, the values of which follow a χ^2^ distribution. Fisher's Exact test was also used as an alternative approach to determine whether outlying families differed in resistance depending on which of the pathogen species was used as inoculum. In calculating the Fisher's Exact test, the equation for the overall regression line was used to adjust the expected values due to the higher infection rate by *M. silenes-inflatae* compared with *M. lagerheimii*. Next, a Bonferroni correction was applied to the Fisher's Exact test to account for the 41 possible independent tests representing all families included in the experiment. Although the Fisher's Exact test uses information from the overall regression to calculate expected numbers of diseased plants, it does not utilize information about the distribution of other families in relation to that regression as the Mahalanobis *D*^2^ approach does.

To help confirm the resistance characteristics of the outlying families (identified as above by the Mahalanobis distances and Fisher's Exact test), a second set of inoculations was performed with the remaining seeds using the methods described above. However, the second experiment was begun in September when greenhouse temperatures were higher leading to lower inoculation success.

## Results

### Field surveys and collections

Anther-smut disease was found in six *S. uniflora* populations among the 14 populations surveyed in 2008; populations used for the inoculation study are indicated in Table 1. Four populations contained only *M. silenes-inflatae*, one population contained only *M. lagerheimii*, and one population from the inland Charterhouse site contained the two *Microbotryum* species in sympatry (Table 1, Fig. 2). The latter population contained 24 specimens of *M. lagerheimii* and one specimen of *M. silenes-inflatae*. Diseased populations tended to be found in the southwestern part of the surveyed region (Fig. 2), although the survey was not exhaustive.

### Variation in physiological resistances

Of the inoculated *S. uniflora* plants (*n* = 3468), 93% flowered and were assessed for disease status. Among families receiving inoculation treatments from both *Microbotryum* species with two pathogen genotypes per species, 25 families had enough flowering plants (*n* ≥ 10) to compare infection rates between *Microbotryum* species. Among families receiving only one pathogen genotype per *Microbotryum* species, 16 families flowered sufficiently to compare infection rates.

**Figure 3:**
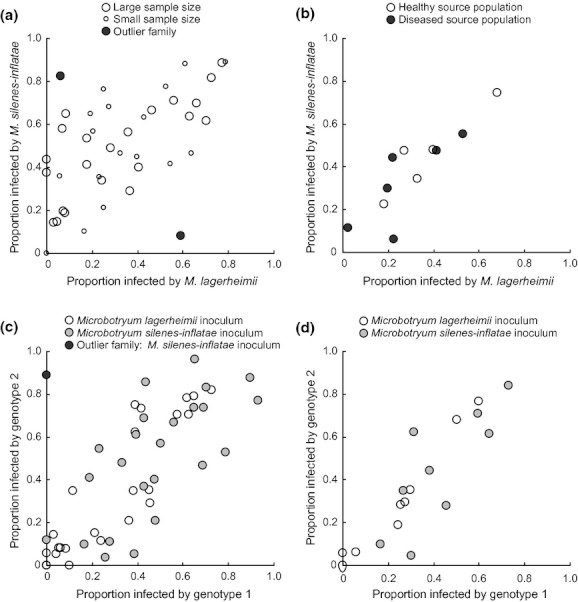
Correlated resistance/susceptibility to anther-smut disease in *Silene uniflora*. (a) Variation in family-level resistance following inoculation with *M. lagerheimii* or *M. silenes-inflatae*. Large and small circles reflect sample size for families that were inoculated with either two (combined) or one *Microbotryum* genotype per species, respectively. Black circles indicate statistical outliers from the overall correlation of resistances, that is, *S. uniflora* families with species-specific resistance. (b) Population-level resistance variation following inoculation with *M. lagerheimii* or *M. silenes-inflatae*. Black circles and white circles indicate whether the source population contained anther-smut disease or not, respectively. (c) Family-level resistance variation following inoculation with different genotypes of *Microbotryum* and separated as to whether the pathogen species was *M. lagerheimii* (white circles) or *M. silenes-inflatae* (gray circles). (d) Population-level resistance variation following inoculation different genotypes of *Microbotryum* and separated as to whether the pathogen species was *M. lagerheimii* (white circles) or *M. silenes-inflatae* (gray circles).

Resistance to *M. lagerheimii* and *M. silenes-inflatae* was significantly positively correlated among all *S. uniflora* populations (Table 2, Fig. 3b). The variance component of infection rates among families within populations was less strongly significant than the among population variance (Table 2, Fig. 3a,b). In particular, in populations found with disease, there was a strong, significant among-family variance contribution to infection rates in contrast to the lack of an among-family component for healthy populations.

**Table 2:** Correlation of resistance to two *Microbotryum* species in *Silene uniflora* populations

	Type of correlation	
	
	Pearson's coefficient	Variance component (SE)
Between *Microbotryum* species		
Among populations		
All populations (*n* = 11)	0.919***	0.849 (0.162)***
Healthy populations (*n* = 5)	0.949**	1.087 (0.168)***
Diseased populations (*n* = 6)	0.773*	0.502 (0.619) ns
Among families within populations		
All populations (*n* = 54)	n.a.	0.305 (0.149)*
Healthy populations (*n* = 26)	n.a.	−0.224 (0.227) ns
Diseased populations (*n* = 28)	n.a.	0.651 (0.138)***
Between *M. silenes-inflatae* genotypes		
Among populations (*n* = 9)	0.980***	n.a.
Between *M. lagerheimii* genotypes		
Among populations (*n* = 9)	0.809**	n.a.

Arcsine transformed data:

* *P* < 0.05,

** *P* < 0.01,

*** ***P < 0.001.

Within each *Microbotryum* species, resistance to different pathogen genotypes was also significantly positively correlated among populations of *S. uniflora* (Table 2, Fig. 3d). Among-family variance components within each *Microbotryum* species, where both strains per species were tested on the same family, were not calculated due to small sample sizes.

Two families (Family 8 from Blakeney Point and Family 6 from Walney Island) deviated from the overall correlation of family-level resistance to *M. lagerheimii* versus *M. silenes-inflatae* (Table 3, Fig 3a). In these two families, the infection rates differed significantly depending upon which pathogen species was used as inoculum according to Fisher's exact test (Table 3), and they were classified as outliers because they had Mahalanobis distances significantly larger than the 95% confidence intervals based upon resistance distributions of the other families (Table 3). Similarly, one family (Family 3 from Walney Island) was classified as an outlier from the correlation of resistance between pathogen genotypes within *M. silenes-inflatae* (Table 3, Fig. 3c).

**Table 3:** Characterization of *Silene uniflora* families with specific resistances to anther-smut disease

	Inoculum = *M. lagerheimii*	Inoculum = *M. silenes-inflatae*	Mahalanobis *P*-value	Fisher's Exact *P*-value^a^
Species-specific resistance				
Blakeney Point – Family 8	6% (*n* = 23)	83% (*n* = 40)	0.019	<0.001
Walney Island – Family 6	59% (*n* = 27)	8% (*n* = 25)	0.008	<0.001

	Inoculum = *M. silenes-inflatae* 1	Inoculum = *M. silenes- inflatae*2	Mahalanobis *P*-value	Fisher's Exact *P*-value^b^
Genotype-specific resistance				
Walney Island – Family 3	0% (*n* = 10)	89% (*n* = 27)	0.011	<0.001

a Includes a Bonferroni correction for 41 independent tests.

b Includes a Bonferroni correction for 25 independent tests.

The characterization of these families as outliers was supported by the second run of inoculations using remaining seeds, although sample sizes were quite limited and greenhouse conditions were not optimal for disease development as described above. In particular, Family 6 from Walney Island had 86% (*n* = 7) of plants diseased by *M. lagerheimii* and 0% (*n* = 6) diseased by *M. silenes-inflatae*; this was a statistically significant confirmation of specific resistance in the repeat inoculations (Fisher's Exact test, *P* = 0.004). The other two repeated tests were not statistically significant, but were each in the same direction as the main experiment. Family 8 from Blakeney Point had 0% (*n* = 12) diseased by *M. lagerheimii* and 4% (*n* = 24) diseased by *M. silenes-inflatae*. Family 3 from Walney Island had 8% (*n* = 12) diseased by genotype 1 of *M. silenes-inflatae* and 15% (*n* = 26) diseased by genotype 2.

## Discussion

The occurrence of multiple pathogens that exploit the same host resource holds the potential to influence both the pathogen community structure and the evolution of host defenses. Across diverse plant and animal systems, our knowledge of these interactions is so limited as to make the suggestion of general patterns difficult. This study provides the first assessment of resistance to anther-smut disease in a host that naturally harbors more than one *Microbotryum* species. While infection rates varied greatly, there was a highly significant pattern of correlated, or “general” resistance among populations of *S. uniflora* to infection by the fungi *M. lagerheimii* and *M. silenes-inflatae*. This result is similar to those found in *Zea mays* (Wisser et al. 2011), *Brassica rapa* (Mitchell-Olds et al. 1995), and *Medicago sativa* (Hill and Leath 1975) for the correlated resistance to multiple fungal pathogens from different genera, at least in some of the pairwise comparisons. In *Microbotryum*, the results suggest that resistance mechanisms that have evolved in response to either pathogen species may often provide protection against the other con-generic pathogen. Resistance that was specific to one pathogen species or genotype was also detected, but much less frequently. Thus, the potential for specific resistances to explain the persistence of both *M. lagerheimii* and *M. silenes-inflatae* on differentially susceptible genotypes of the host *S. uniflora* appears unlikely.

Sympatry of *M. lagerheimii* and *M. silenes-inflatae* in populations of *S. uniflora* was not frequent among the sites surveyed in this study, but prior research has shown the co-occurrence of these two *Microbotryum* species on *S. vulgaris* in the Alps (Bucheli et al. 2000; Le Gac et al. 2007a). Only the Charterhouse population of *S. uniflora* contained both *Microbotryum* species, and this was represented only by a single sample of *M. silenes-inflatae* among 24 *M. lagerheimii* samples. Sympatry of *M. lagerheimii* and *M. silenes-inflatae* has also been observed in *S. uniflora* populations in Suffolk, England (E. Petit, J. Watson, P. Gladieux, T. Giraud, J. Antonovics, A. Pedersen, M. E. Hood, unpubl. data), but seed collections were not available to assess their resistance characteristics. Existing theory suggests that fixed versus variable frequencies of encounters with each individual pathogen species would result in specialized versus generalized resistance, respectively (Lapchin 2002). On the basis of the patterns of pathogen sympatry in the sampled populations, we may have expected specific resistance to be more common. However, we found the opposite pattern, that while there is great variation in the level of resistance among families, for most families, resistance provides similar levels of protection for the two pathogens. Further studies are needed to determine whether sympatry of multiple *Microbotryum* species in a single population is a transient occurrence, whether it is facilitated by factors other than specialized resistance in the host, or whether the evolution of resistance specificities is less likely in populations with sympatric pathogen species. Although the history of disease in the populations that were studied is unknown, it was interesting that the resistances among families were more strongly correlated in families collected from populations that were currently diseased; this supports the idea that selection for resistance to one pathogen species results in resistance to the other species. However, it should also be noted that besides a history of selection, there remains the possibility that maternal effects contributed to the assessment of resistance and that diseased populations may also be geographically separated in latitude from healthy populations.

The occurrence of general resistance, combined with strong evidence for specific resistance in a minority of the families, raises questions about the genetic determination of resistance. This has not been investigated. There remains the possibility that maternal effects contributed to the assessment of resistance, and this cannot be excluded. It is also possible that generalized resistances might be more important under the laboratory inoculation procedure that has been optimized to achieve a high infection success. Studying specificity in field conditions should therefore be a high priority in future studies.

If resistance to anther smut in the field is indeed general resistance, then this has important implications for the distribution of the species of *Microbotryum*. First, resistance to an endemic *Microbotryum* species may protect the host from the emergence via host shifts of related pathogens by lowering both the density and proportion of susceptible individuals. We have previously found evidence for host shifts of anther-smut disease into disease-free populations of *S. vulgaris* in North America and Europe (Hood et al. 2003). The susceptible genotypes may thus be particularly prone to receive the disease as host shifts from another species. In this system, there was substantial among family variation in cross-species transmission (Antonovics et al. 2002). Similarly, for a host with multiple endemic *Microbotryum* species, the local history of exposure and resistance evolution, could determine conditions limiting invasion by immigrant pathogens. A recent study has revealed wide variation in the prevalence of anther-smut disease among *Silene* species (Hood et al. 2010), and therefore it would be informative to assess the levels and generality of anther-smut resistance across this group of hosts and whether this influences the success of cross-species disease transmission. Studies on parasitoids of insects and other fungi on plants (Mitchell-Olds et al. 1995; Fellowes et al. 1999) have also shown the presence of generalized cross-species resistance, but the evolutionary implications for disease emergence have yet to be thoroughly explored.

The second major implication of general resistance is that co-occurring pathogen species may compete more directly for a common set of susceptible host genotypes than if there were species-specific resistances, perhaps increasing the likelihood of co-infection and the within-host dynamics that drive pathogen virulence strategies (van Baalen and Sabelis 1995; May and Nowak 1995). Several studies have addressed co-infection dynamics in the anther-smut system, with indications that the strength of within-host competition is influenced by pathogen relatedness (Hood 2003; Koskella et al. 2006; López-Villavicencio et al. 2007, 2011). In particular, Gold et al. (2009) showed that the competitive exclusion between *M. lagerheimii* and *M. silenes-inflatae* was stronger than exclusionary interactions between genotypes within either species. In both Gold et al. (2009) and the current study, there was also a difference in infection ability between the *Microbotryum* species, even though different pathogen isolates and hosts were used; *M. silenes-inflatae* caused relatively higher infection rates than *M. lagerheimii*. Inoculations in the present experiment were carried out with each pathogen genotype applied singly. However, whether the competitive dynamics of multiple infections might interact with the observed variation in susceptibility in *S. uniflora*, including species-specific resistances, should be investigated in further studies.

It was surprising that no disease was found in the more northern populations on either the western or eastern coasts of England. The presence of disease in the southeastern coast is known from natural history collections, as well as ongoing studies of diseased populations in Suffolk and Kent (E. Petit, J. Watson, P. Gladieux, T. Giraud, J. Antonovics, A. Pedersen, M. E. Hood, unpubl. data). It is particularly interesting that instances of pathogen-specific resistance were found in *S. uniflora* families from the healthy northern populations. However, the current distribution of *Microbotryum* may not fully inform the history of selection for resistance and the “ghosts of disease past.” In fact, a century ago Sir Edward J. Salisbury noted the presence of anther-smut disease on *S. uniflora* at Blakeney Point in the county of Norfolk (Salisbury 1912), a population now containing species-specific resistance to *M. lagerheimii* in one family, but where in our current survey, a thorough search of the site found no disease.

In the absence of strong and widespread patterns of specific resistances, other explanations should be sought to explain the co-occurrence of *M. lagerheimii* and *M. silenes-inflatae* on *S. uniflora*. For example, we have not studied timing of disease expression nor possible differences in micro-climate effects on the transmission or infection success of the two pathogen species. Given the rare co-occurrence of the two species in natural populations, the histories of migration and colonization by the multiple *Microbotryum* species on *S. uniflora* and *S. vulgaris* may help explain the distributions of multiple pathogens on these host species; studies of related species of *Microbotryum* on other host species have shown phylogeographical effect to be important (e.g., Vercken et al. 2010). With increased ability to identify often cryptic differences between pathogen species and the growing impact of anthropogenic dispersal worldwide, such studies can provide important insights into the determinants of modern community assemblages.
